# Comparison of the Effects of Oral Hygiene Instruction Methods on Oral Hygiene and Self-Perception in Older Adults: A Randomized Controlled Trial

**DOI:** 10.3390/jcm13247642

**Published:** 2024-12-15

**Authors:** Inês Caetano Santos, Catarina Colaço, Giancarlo De la Torre Canales, Luís Proença, Mário Polido, José João Mendes, Helena Canhão, Ana Cristina Manso

**Affiliations:** 1 Egas Moniz Center for Interdisciplinary Research (CiiEM), Egas Moniz School of Health & Science, 2829-511 Almada, Portugal; ccolaco@egasmoniz.edu.pt (C.C.); gcanales@egasmoniz.edu.pt (G.D.l.T.C.); lproenca@egasmoniz.edu.pt (L.P.); mpolido@egasmoniz.edu.pt (M.P.); jmendes@egasmoniz.edu.pt (J.J.M.); cmanso@egasmoniz.edu.pt (A.C.M.); 2 NOVA Medical School, Faculdade de Ciências Médicas, Universidade Nova de Lisboa, 1169-056 Lisbon, Portugal; helena.canhao@nms.unl.pt; 3 Comprehensive Health Research Center (CHRC), Universidade Nova de Lisboa, 1169-056 Lisbon, Portugal; 4 Division of Oral Diagnostics and Rehabilitation, Department of Dental Medicine, Karolinska Institutet, SE-14104 Huddinge, Sweden; 5 Department of Dentistry, Ingá University Center, Uningá, Paraná 87035-510, Brazil; 6 LA REAL, ULS São José, 1900-028 Lisbon, Portugal

**Keywords:** oral health, oral hygiene, older, health motivation, self-assessment

## Abstract

**Background:** Age-related conditions, such as being misinformed, having limited oral health literacy, and the loss of manual dexterity, autonomy, or visual acuity, may act as barriers to oral health. The aim of this study was to evaluate the effectiveness of two different oral hygiene instruction methods on oral hygiene and the self-perception of oral health in older adults. **Methods:** This randomized controlled trial included participants aged 65 and older who completed a questionnaire on socio-economic factors, self-perceived oral health, and oral hygiene behaviours. Oral hygiene status was assessed using the Oral Hygiene Index—Simplified (OHI-S). Participants were randomly allocated into two different groups, according to the method of oral hygiene instruction: a “General Approach” (GA) (*n* = 28) and a “Personalized Technique” (PT) (*n* = 26). After two months, a follow-up session was conducted. Data were analysed using descriptive and inferential methodologies. **Results:** The GA and PT methods were effective in promoting oral hygiene behaviours, with a significant increase in the use of interdental devices, but no significant differences were found between the two methods. Self-perceived oral health did not change significantly, neither after the instruction nor between methods. Significant improvements were achieved with both methods for the OHI-S, with significant differences between the two methods for the Calculus Index, where the PT achieved better results. **Conclusions:** Oral hygiene education leads to improvements in the adoption of oral hygiene behaviours and clinical indicators. Furthermore, a personalized approach promoted better results in clinical indicators.

## 1. Introduction

The demographic transition to an ageing society has become a global challenge, with the number of older people expected to increase to 1.5 billion, 16.0% of the total population, by 2050 [1,2]. Despite this rapid worldwide growth, too-limited attention has been given to how older people live, what and how they can contribute to society, and what services and support they may need. Regarding the ageing population, the world remains inadequately prepared to meet the challenges and opportunities of global demographic change [3].

In terms of oral health, there is an increased susceptibility to oral diseases in this age group, such as dental caries and periodontal disease, which can lead to tooth loss, affecting aesthetics, phonetics, and chewing function. Poor oral health also affects overall health, well-being, and quality of life, and oral diseases have been associated with some chronic diseases, such as diabetes and cardiovascular and respiratory diseases [4,5,6,7]. In addition, while there are structural factors that promote oral health inequalities, leading to financial constraints on treatment and limited access to oral health care, there are also individual factors, often age-related, that can act as barriers to good oral health, such as misinformation or limited oral health literacy and the loss of manual dexterity, autonomy, or visual acuity, which can lead to some neglect of oral hygiene in terms of toothbrushing and interdental cleaning and oral care concerns [7,8,9,10]. This highlights the importance of improving oral health care for vulnerable older adults.

The maintenance of oral health, which is directly related to proper oral hygiene, is highly dependent on two factors: the patient’s motivation/cooperation and the patient’s ability to perform oral hygiene effectively. Given that bacterial plaque can be effectively removed by proper oral hygiene, there is a need to enhance oral hygiene behaviours and to invest in education and motivation with appropriate oral health interventions that consider the specific needs of this ageing group [11,12]. Oral health education promotes the improvement of general knowledge, which may lead to the adoption of favourable oral health behaviours, which in turn could contribute to a reduction in the prevalence of oral diseases [13].

The results of oral health education can be measured by objective clinical measures of oral health, but self-perceived oral health is a subjective measure that has been shown to be associated with clinical indicators and psychosocial factors and should, therefore, be considered a complementary indicator [14,15,16]. In addition, subjective measures of oral health involve multiple factors, not only personal experiences but also the environment and social context. In older adults, some studies reported that their self-perception of health is negative, while others report a good self-perception, even with poor oral health [14,15,16,17,18,19]. It is, therefore, important to assess whether oral health self-perception is modified by education techniques [20]. Notwithstanding this, there is still no consensus on which factor has the greatest impact on improving oral hygiene behaviours, as several studies have tested different theory-based behavioural models, but all of them point to the importance of education and subjective indicators of oral health in patients’ compliance with oral hygiene behaviours [21,22,23].

Although some studies have been conducted on the importance of oral health instruction methods, the literature suggests that the results are still insufficient and that there is a need to implement effective oral health programs that ensure the involvement of the older population to fully assess their impact on oral health [13,24]. In addition, self-perceived oral health has not been included in the assessment of post-intervention changes in previous studies. Furthermore, behavioural studies in older populations are important for introducing or modifying oral health policies to address oral health barriers in this age group [21,22,25]. Thus, the aim of this study was to evaluate the effectiveness of two different oral hygiene instruction methods on oral hygiene and self-perception of oral health in older adults.

## 2. Materials and Methods

This two-armed randomized controlled trial (RCT) was conducted at a university dental hospital (Egas Moniz Dental Clinic, Almada, Portugal) from 30 January 2023 to 29 May 2023. This study was approved by the Ethics Committee of the Egas Moniz School of Health & Science, Portugal (No. 1131 of 26 January 2023), registered at ClinicalTrials.gov (NCT06444490) and conducted according to the tenets of the Declaration of Helsinki. All participants were thoroughly informed about the aims of this study and provided written consent to participate in the clinical trial. The reporting of data followed the CONSORT checklist [26].

### 2.1. Participants

A total of 60 participants were recruited as a convenience sample at Egas Moniz Dental Clinic. Inclusion criteria were being over 65 years of age, not being totally edentulous, not being institutionalized, and being able to understand and sign an informed consent form. Participants also had to be able to speak and understand Portuguese, be literate, and be able to comply with the study protocol, i.e., not have disabilities such as blindness, deafness, or dementia.

### 2.2. Study Protocol, Randomization, and Blinding

**Figure 1:**
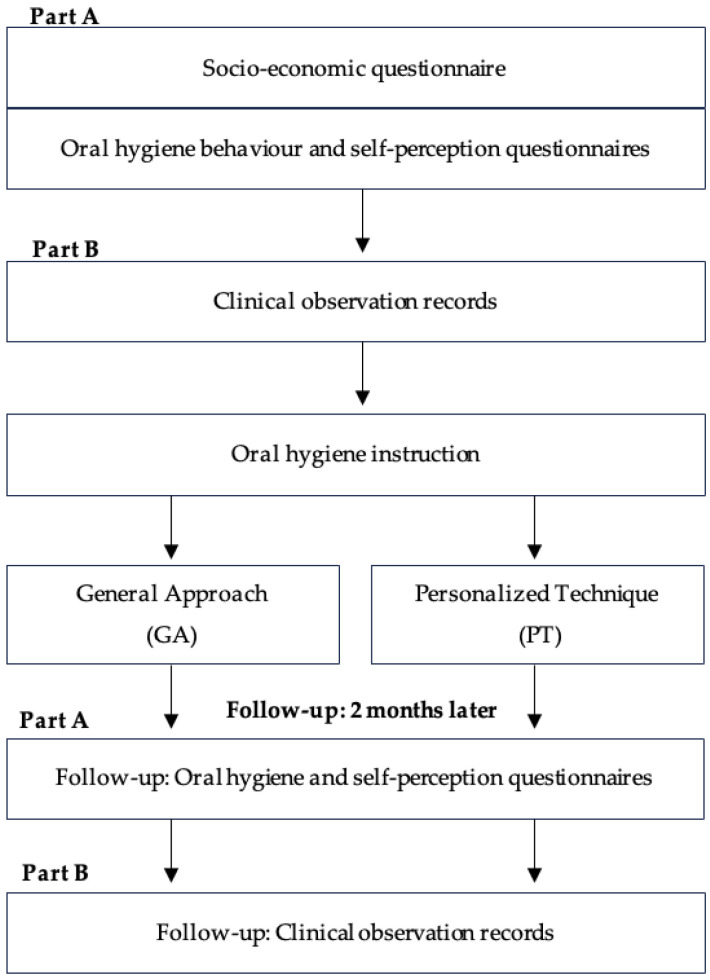
Study flow diagram.

In this study, participants completed a questionnaire focusing on socio-economic data, oral hygiene behaviours, and self-perceived oral health (Part A) at baseline. Clinical parameters of participants’ oral hygiene were then obtained by intraoral examination (Part B). At this stage, oral hygiene instruction was provided (“General Approach (GA)” or “Personalized Technique (PT)”), depending on the instruction method assigned. Two months later, a follow-up session was conducted. The study flow diagram is shown in Figure 1.

For the oral hygiene instruction, participants were randomly allocated into two groups, with 30 participants (*n* = 30) in each group, and exposed to different methods of professional education. A computer-generated random number list with allocation concealment was used to assign participants to 1 of the 2 groups (1:1 proportion). This allocation was kept in a sealed envelope, which was not opened until the instruction session. The randomization was performed by an external investigator, not involved in the intervention or assessment. In addition, the researcher responsible for outcome assessment was blinded to intervention allocation.

### 2.3. Intervention

For oral hygiene instruction, we defined two groups: The first group, identified as the “General Approach” (GA) group, focused more broadly on the different topics of oral diseases and oral hygiene care. Instruction for the second group, identified as the “Personalized Technique” (PT), was based on the specific needs of each participant and was considered the “Tell-Show-Do” method [27]. All oral hygiene instructions were performed by the same dentist in both groups, using a checklist to ensure that the same procedures and topics were covered.

Instruction for the first group, GA, consisted of a 15 min single session in which the dentist attempted to impart knowledge about oral hygiene in general, highlighting some of the most common conditions in this age group, namely the difficulty in achieving correct brushing and interdental cleaning, demonstrating the correct technique on a typodont (model), and alerting them to the importance of oral health to systemic health. Periodontal disease, dental caries and denture care were also covered in general terms. Participants were then given a written explanatory guide to reinforce good oral hygiene habits and an explanation of these diseases. Oral hygiene tools were also provided to encourage the expected improvement in oral health. Finally, there was a question-and-answer session to clarify any doubts the participant might have.

As for the method applied to the second group, the PT is more patient-centred. This approach began with a self-examination using an extraoral mirror by the participant to allow the professional to understand each participant’s perception of their oral health and their concerns, and the use of a plaque-disclosing solution to allow the participant to be more sensitive to the areas where brushing and interdental cleaning were not as effective. After understanding the participant’s oral hygiene behaviour, the aim was to alert them to the most inappropriate aspects of their oral hygiene and to explain and define the best solutions to adopt to improve it, adapting the oral hygiene tools to their clinical conditions. At this stage, conditions such as dental caries, major gingival recessions, areas of greater inflammation, areas where dental plaque accumulation or even tartar was more visible (both in the mouth and on the denture), xerostomia symptoms, and further denture care were highlighted. The typodont (model) was then used to demonstrate the correct toothbrushing and interdental cleaning techniques, allowing participants to practice brushing and cleaning interdental spaces until they were proficient. In the case of periodontal disease and dental caries, an explanation of these diseases and the benefits of good oral hygiene in preventing their rapid progression was given. The session ended in the same way as the first method, with the participant receiving an explanatory guide and more appropriate tools to promote the expected improvement in oral health. In addition, a personalized plan was created for the participant to take home to improve their oral hygiene techniques. The same question-and-answer session was used to clarify any doubts. This PT method consisted of a single session of approximately 20 min.

### 2.4. Outcomes

Participants’ outcomes were assessed at two time points (baseline and 2 months) over the 2-month study period. Both subjective and objective outcomes were conducted at each evaluation period by a researcher not involved in any other study procedures. For the subjective assessment, a structured questionnaire about socio-economic data, oral hygiene behaviours, and self-perceived oral health was used, while oral hygiene clinical indicators were the objective assessments. The primary outcomes of this study were oral hygiene behaviours and self-perceived oral health. The secondary outcomes were clinical oral hygiene indices (DI-S, CI-S, and OHI-S).

#### 2.4.1. Part A—Socio-Economic Data, Oral Hygiene Behaviours, and Self-Perceived Oral Health

A self-reported structured questionnaire collected information on socio-economic data, including age (years), sex (female/male), whether participants lived alone or not, education level, categorized as primary school or less (≤4 years), or more than primary school (>4 years), and monthly income (<800 EUR/≥800 EUR).

The oral hygiene behaviours questionnaire included brushing frequency (<2x/day/≥2x/day) and use of interdental devices (“Do you use an interdental brush or dental floss?”, with yes/no responses). For denture users, the questions surveyed about denture hygiene and care were “Do you clean your denture every day?”; “Do you usually sleep with your dentures on?”, with dichotomous (yes/no) responses.

Self-perceived oral health was assessed through the following questions: “How would you describe your oral health?”, “How would you describe the condition of your teeth?”, and “How would you describe the condition of your gums?”. The answers to the questions were classified as poor (“neither satisfied nor unsatisfied”, “unsatisfied”, and “very unsatisfied”) or good (“very satisfied” and “satisfied”) [14]. For xerostomia assessment, the survey question was “Do you feel your mouth dry: yes/no?”.

#### 2.4.2. Part B—Oral Hygiene Clinical Indicators

Clinical recordings were collected by a single, blinded, and experienced general dentist who had previously been subjected to a calibration procedure on ten patients not included in the study. Measurement reliability and reproducibility were assessed by the intra-class correlation coefficient (ICC), and the intra-examiner agreement was 0.96. The room used for the observations had both natural and artificial lighting. The equipment used included an intraoral mirror, a CPI probe, gloves, a mask, and compresses [28]. Data on the Oral Hygiene Index—Simplified (OHI-S), which is the sum of the arithmetic mean of the Debris Index—Simplified (DI-S) and the Calculus Index—Simplified (CI-S), could be obtained from the clinical records. We then applied the terms “good”, “fair”, and “poor” to correspond to selected levels of debris and calculus as follows: 0.0 to 0.6 is considered good, 0.7 to 1.8 is deemed fair, and 1.9 to 3.0 is considered poor, with the OHI-S value being the sum of the two: 0.0 to 1.2 (good), 1.3 to 3.0 (fair), and 3.1 to 6.0 (poor) [29].

### 2.5. Data Analysis

Statistical analyses were performed using IBM SPSS Statistics v.29 software and included descriptive and inferential methodologies. For descriptive analysis, categorical data were presented as frequency and percentage distributions and numerical data as mean and standard deviation (SD). Inferential methodologies included the application of statistical tests, depending on the characteristics of the variables and the type of comparison (Chi-squared test/Fisher’s exact test, McNemar test, Student’s *t*-tests for independent and paired samples). Effect sizes were identified by calculating Cohen’s d and Cramer’s V values. A significance level of 5% (*p* ≤ 0.05) was established in all inferential analyses.

## 3. Results

**Figure 2:**
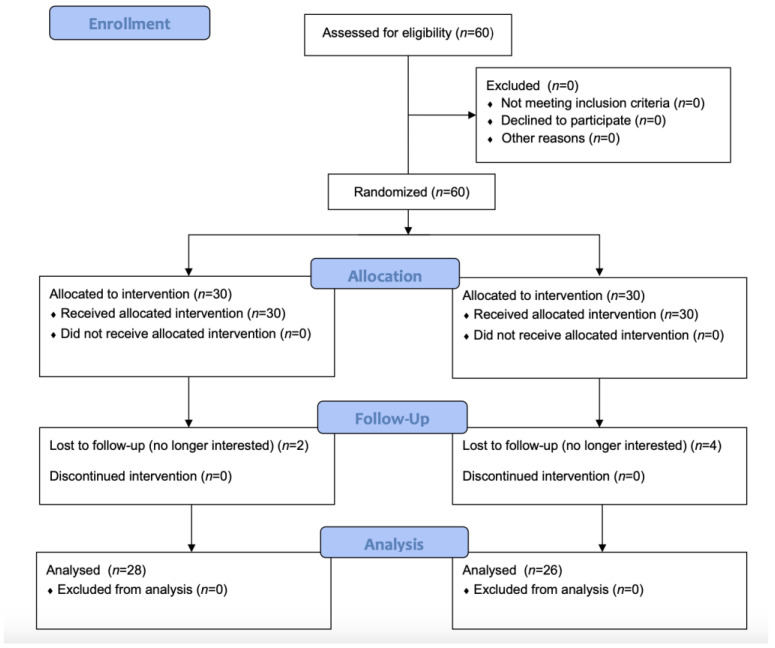
RCT study CONSORT diagram.

A total of 60 participants were assessed for eligibility. Of these, six participants were lost to follow-up. For the GA, 28 participants completed the follow-up, and for the PT, 26 participants completed this assessment (Figure 2).

### 3.1. Socio-Economic Characteristics of the Study Participants

Participant characteristics regarding the socio-economic variables are shown in Table 1. Inter-group comparisons showed that the GA group was older (t(df = 53) = 2.021, *p* = 0.048) and had a lower level of education (χ2(df = 3) = 8.620, *p* = 0.003) compared to the PT group. No significant differences were found in other socio-economic variables.

### 3.2. Oral Hygiene Behaviours

Regarding intra-group comparisons and considering oral hygiene behaviours, only the use of an interdental device showed statistically significant improvements between baseline and follow-up in the two instruction method groups (GA: χ2(df = 3) = 5.143, *p* = 0.016; PT: χ2(df = 3) = 4.167, *p* = 0.031). Considering inter-group comparisons, no statistically significant differences were found between the two methods of instruction (Table 2).

### 3.3. Self-Perception of Oral Health

Regarding self-perception of oral health, no significant differences were found for both instruction methods between baseline and follow-up assessments in intra-group comparisons, nor were any found between the two instruction methods (Table 3).

### 3.4. Oral Clinical Indicators

Considering intra-group comparisons, the DI-S (GA: t(df = 27) = 8.131, *p* < 0.001; PT: t(df = 25) = 5.447, *p* < 0.001), CI-S (GA: t(df = 27) = 2.299, *p* = 0.029; PT: t(df = 25) = 4.750, *p* < 0.001), and OHI-S (GA: t(df = 27) = 7.485, *p* < 0.001; PT: t(df = 25) = 5.691, *p* < 0.001) (*p* < 0.001) baseline scores decreased at follow-up for both instruction methods (Table 4).

Regarding inter-group comparisons, we found no statistically significant differences between the two methods for DI-S and OHI-S scores. However, a statistically significant difference was observed for the CI-S (t(df = 53) = 2.804, *p* = 0.007), with a higher reduction in the PT method (Table 4).

## 4. Discussion

To the best of our knowledge, this is the first study conducted in Portugal to assess the effectiveness of two instruction methods on subjective and objective indicators of oral hygiene among older adults. Our findings showed that both instruction methods applied in this study increased the use of interdental cleaning devices, although there were no differences between the methods. However, the PT method obtained significantly better results for the CI-S improvements, even though both methods improved all clinical indicators.

Despite the scientific consensus that brushing is the most effective method of removing plaque, empirical evidence suggests that brushing alone may only remove up to 60% of total plaque during each cleaning session [30]. Additionally, several reports suggest that brushing is more effective at cleaning the facial surfaces of teeth and less effective on the interdental surfaces. This finding is significant because interdental areas are at the highest risk for plaque accumulation on both anterior and posterior teeth [30,31]. Therefore, interdental cleaning devices play a crucial role in the prevention of oral diseases and conditions, such as gingivitis, coronal and interproximal caries, and tooth loss [12,32]. In our study, dental floss and interdental brushes were evaluated collectively, which may have contributed to higher baseline adherence levels than those reported in most studies [33,34,35], as patient compliance with daily flossing is typically low, largely due to a lack of motivation or difficulties in using dental floss, especially among older adults who may experience decreased dexterity and motor skills [30]. However, the use of interdental brushes is considered to be the most effective for interdental cleaning due to greater ease of use, better ergonomic handlers, and patient acceptance [30]. Therefore, demystifying the existence of easier-to-use interdental cleaning devices appears to be sufficient for patient compliance, which may explain the success of both methods without favouring one over the other.

Despite some variation in reported daily denture care practices in the literature [33,36,37], they tend to be generally high and favourable, aligning with this study’s findings and potentially explaining the lack of significant differences between groups. The widespread practice of denture care can be attributed to its accessibility, simplicity, and affordability [33,36]. Moreover, proper denture cleaning is crucial for maintaining good oral health, as it helps prevent unpleasant odours, maintains aesthetic appearance, and prevents the accumulation of dental plaque and calculus, which can lead to oral mucosal lesions that affect overall health, particularly in older adults [38]. It is also recommended to remove dentures when sleeping to prevent stomatitis caused by microorganisms, particularly *Candida albicans*, or by trauma [36,37]. Therefore, education about denture care practices remains essential in daily clinical practice.

Regarding self-perceived oral health, contrary to our results, some studies suggest that knowledge of oral hygiene practices and oral health literacy can impact how patients perceive their oral health [14]. However, it is interesting to note that the perception of gum health in this study showed a tendency to achieve better results after both instruction methods, which could be attributed to better oral hygiene, often associated with a reduction in gingival inflammation [12,22], increasing the perception of gum health.

When examining clinical oral indicators, the PT method obtained significant and the most favourable results for the CI-S. This finding could be because a more individualized approach allowed participants to visibly recognize the accumulation of calculus, which was more apparent than debris, potentially influencing their attitudes towards visiting the dentist for a check-up or professional tooth cleaning. However, it is important to note that participants in the GA group were significantly older and had lower levels of education. These factors might have affected the effectiveness of this instructional method, since older age is often associated with reduced autonomy and dexterity, and lower education levels are linked with reduced oral health literacy, which can be a barrier to maintaining proper oral hygiene practices [9,10,39,40]. Moreover, we observed significant improvements in all oral hygiene clinical indices for both instruction methods at follow-ups, indicating the success and effectiveness of both interventions. Regular interdental cleaning is known to reduce dental plaque, calculus, and gingivitis [30]. Thus, significant improvements in clinical indicators are likely to result from the substantial adherence to interdental cleaning devices found in this study.

As a final note, this study’s results indicate that oral hygiene instruction can improve the oral hygiene of older adults, regardless of the method used. This underscores the importance of motivating older adults during dental visits and through educational programs. However, the PT method showed slightly better outcomes, suggesting that personalized and detailed attention enhances results. Nevertheless, the improvements in the GA group demonstrate that benefits can also be achieved through training, lectures, workshops, or even teledentistry. Our results aim to contribute to future national public oral health strategies by demonstrating the importance of expanding oral health motivational and educational interventions to ensure improvements in oral health for older adults. Furthermore, in countries with still-low public sector coverage of dental services and with unequal geographical distributions of oral health care, such as Portugal, preventive dentistry focused on education could help reduce dental treatment costs and inequalities in the burden of oral diseases.

### Limitations

This study is not free from limitations. In the present study, the small sample size and the fact that it was a convenience sample limit the generalizability of our findings and our ability to detect minor effects. However, this did not prevent our study from obtaining statistically significant differences in some parameters within and between groups. In addition, we performed an a posteriori power analysis to confirm the adequacy of the sample size. Using the CI-S as a clinical outcome, considering the differences between groups after follow-up, a Cohen’s d effect of 0.78 was determined. According to this value, to establish an alpha error of 5% and a power of 80%, a total sample size of 52 participants (*n* = 26 per group) was required. The follow-up period of this study may also be too short to show more robust results. However, we consider it important that this evaluation in this specific population be shorter, so that we do not lose contact with the study participants. Future research on this topic should involve a larger population and a longer period of time. Therefore, this study could be a basis for planning such trials, as there are no studies in the Portuguese elderly population involving oral health education. Another limitation is that the participants in the GA group were significantly older and had lower levels of education than those in the PT group, which may have influenced the more positive results in the latter group. In addition, we did not have a control group without intervention. There may also have been some sample bias due to the fact that this population attends a university clinic, which is often motivated by dental students, and this may have influenced the results. Finally, this study did not consider participants’ oral health literacy before and after the intervention and did not include a cognitive–behavioural approach in the intervention evaluation. Further studies could include these aspects when assessing the attitudes and perceptions of older adults.

## 5. Conclusions

The study results suggest that both the GA and PT instruction methods effectively promote improvements in using interdental cleaning devices and oral hygiene score levels in older adults, with a personalized approach potentially maximizing the clinical outcomes. However, self-perceived oral health did not change significantly either after the instruction method or between methods.

As the global older population continues to grow and life expectancy increases, it is becoming increasingly important to invest in preventative oral and systemic health measures in these patients. A more personalized approach through regular dental visits would be ideal, but a generalized approach could also be considered to improve oral hygiene in this population, providing education and literacy to enable the patient to adopt appropriate behaviours and, thus, prevent potential oral diseases. This generalized approach could be further evaluated in more detail, and, if proven successful and cost-effective in a wider range of studies, it could be useful for national oral health strategies aiming to include older adults with less accessibility to oral health care services.

## Figures and Tables

**Table 1:** Socio-economic characteristics and denture use of study participants.

		Total (*n* = 54)	GA (*n* = 28)	PT (*n* = 26)	*t* Value *	χ2 Value *	Effect Size *	*p*-Value *
Age (years), mean (± SD)		73.2 (±6.6)	74.9 (±6.6)	71.4 (±6.2)	2.021	-	6.44 ^a^	0.048
Sex, *n* (%)	Female	23 (42.6)	12 (42.9)	11 (42.3)	-	0.002	0.006 ^b^	0.967
	Male	31 (57.4)	16 (57.1)	15 (57.7)				
Living alone, *n* (%)	No	37 (68.5)	19 (67.9)	18 (69.2)	-	0.012	0.015 ^b^	0.914
	Yes	17 (31.5)	9 (32.1)	8 (30.8)				
Education level, *n* (%)	≤4 years	19 (35.2)	15 (53.6)	4 (15.4)	-	8.620	0.40 ^b^	0.003
	>4 years	35 (64.8)	13 (46.4)	22 (84.6)				
Monthly income (EUR), *n* (%)	<800	7 (13.0)	6 (21.4)	1 (3.8)	-	-	-	-
	≥800	38 (70.3)	17 (60.7)	21 (80.2)				
	NA	9 (16.7)	5 (17.9)	4 (15.4)				
Denture use, *n* (%)	Yes	51 (94.4)	26 (92.9)	25 (96.2)	-	0.279	0.07 ^b^	0.597
	No	3 (5.6)	2 (7.1)	1 (3.8)				

* GA vs. PT (Chi-squared (χ2) test), except for age (Student’s *t*-test for independent samples). Effect size: ^a^ Cohen’s d; ^b^ Cramer’s V. Abbreviations: GA—General Approach; *n*—number of participants; NA—not answered; PT—Personalized Technique; SD—standard deviation.

**Table 2:** Changes in oral hygiene behaviours (baseline vs. follow-up) according to instruction method and comparison of oral hygiene behaviours between the two instruction methods.

		GA (*n* = 28)			PT (*n* = 26)				
		Baseline		Follow-Up	Baseline		Follow-Up	χ2 Value **	*p*-Value **
Brushing frequency, *n* (%)	<2x/day	24 (85.7)		1 (3.6)	4 (15.4)		1 (3.8)	0.090	1.000
	≥2x/day	4 (14.3)		27 (96.4)	22 (84.6)		25 (96.2)		
	χ2 value *		0.800			1.333			
	*p*-value *		0.375			0.250			
Interdental device, *n* (%)	No	13 (46.4)		6 (21.4)	10 (38.5)		4 (15.4)	0.027	0.869
	Yes	15 (53.6)		22 (78.6)	16 (61.5)		22 (84.6)		
	χ2 value *		5.143			4.167			
	*p*-value *		0.016			0.031			
Daily denture hygiene, *n* (%)	No	6 (23.1)		1 (3.8)	0 (0.0)		0 (0.0)	-	-
	Yes	20 (76.9)		25 (96.2)	25 (100.0)		25 (100.0)		
	χ2 value *		2.286			-			
	*p*-value *		0.125			-			
Sleeping with denture, *n* (%)	No	23 (88.5)		25 (96.2)	20 (80.0)		25 (100.0)	0.690	0.465
	Yes	3 (11.5)		1 (3.8)	5 (20.0)		0 (0.0)		
	χ2 value *		0.250			-			
	*p*-value *		0.625			-			

* Follow-up vs. baseline (McNemar test). ** GA vs. PT (Chi-squared (χ2) test/Fisher’s exact test). Abbreviations: GA—General Approach; *n*—number of participants; PT—Personalized Technique.

**Table 3:** Changes in self-perception (baseline vs. follow-up) according to instruction method and comparison of self-perception between the two instruction methods.

		GA(*n* = 28)			PT (*n* = 26)				
		Baseline		Follow-Up	Baseline		Follow-Up	χ2 Value **	*p*-Value **
Oral health perception, *n* (%)	Good	24 (85.7)		23 (82.1)	23 (88.5)		22 (84.6)	0.003	1.000
	Poor	4 (14.3)		5 (17.9)	3 (11.5)		4 (15.4)		
	χ2 value *		0.000			0.000			
	*p*-value *		1.000			1.000			
Teeth perception, *n* (%)	Good	24 (85.7)		24 (85.7)	24 (92.3)		25 (96.2)	0.436	0.604
	Poor	4 (14.3)		4 (14.3)	2 (7.7)		1 (3.8)		
	χ2 value *		0.000			0.000			
	*p*-value *		1.000			1.000			
Gum perception, *n* (%)	Good	24 (85.7)		26 (92.9)	24 (92.3)		25 (96.2)	0.147	1.000
	Poor	4 (14.3)		2 (7.1)	2 (7.7)		1 (3.8)		
	χ2 value *		0.167			0.000			
	*p*-value *		0.625			1.000			
Xerostomia, *n* (%)	No	16 (57.1)		13 (46.4)	10 (38.5)		9 (34.6)	0.436	0.604
	Yes	12 (42.9)		15 (53.6)	16 (61.5)		17 (65.4)		
	χ2 value *		0.800			0.000			
	*p*-value *		0.375			1.000			

* Follow-up vs. baseline (McNemar test). ** GA vs. PT (Chi-squared (χ2) test/Fisher’s exact test). Abbreviations: GA—General Approach; *n*—number of participants; PT—Personalized Technique.

**Table 4:** Changes in clinical indicators (baseline vs. follow-up) according to instruction method and comparison between the two instruction methods (GA vs. PT).

	Instruction Method	Baseline (b)	Score	Follow-Up (f)	Score	Diff. (f–b)	*t* Value *	Effect Size *	*p*-Value *	*t* Value **	Effect Size **	*p*-Value **
DI-S, Mean (±SD)	GA	0.95 (±0.47)	Fair	0.38 (±0.37)	Good	−0.56 (±0.37)	8.131	0.37	<0.001	−0.526	0.42	0.601
	PT	0.95 (±0.47)	Fair	0.45 (±0.34)	Good	−0.50 (±0.47)	5.447	0.47	<0.001			
CI-S, Mean (±SD)	GA	0.35 (±0.41)	Good	0.23 (±0.30)	Good	−0.12 (±0.27)	2.299	0.27	0.029	2.804	0.35	0.007
	PT	0.55 (±0.41)	Good	0.16 (±0.21)	Good	−0.39 (±0.41)	4.750	0.41	<0.001			
OHI-S, Mean (±SD)	GA	1.30 (±0.71)	Fair	0.61 (±0.59)	Good	−0.68 (±0.48)	7.485	0.48	<0.001	1.162	0.65	0.250
	PT	1.50 (±0.83)	Fair	0.61 (±0.45)	Good	−0.89 (±0.80)	5.691	0.79	<0.001			

* Follow-up vs. baseline (Student’s *t*-test for paired samples). ** Difference (f–b): GA vs. PT (Student’s *t*-test for independent samples). Effect size: Cohen’s d. Abbreviations: b—baseline; CI-S—Calculus Index—Simplified; Diff. (f–b)—difference between follow-up and baseline score values; DI-S—Debris Index–Simplified; f—follow-up; GA—General Approach; OHI-S—Oral Hygiene Index—-Simplified; PT—Personalized Technique; SD—standard deviation.

## Data Availability

The entirety of the data examined throughout this study can be found in this document. The datasets analysed in the current study are available from the corresponding author upon reasonable request.
